# Harmful Algal Bloom Monitoring with Unmanned Aerial Vehicles: Tools, Challenges, and Public Health Implications

**DOI:** 10.3390/toxins17100475

**Published:** 2025-09-24

**Authors:** Kendall Byrd, Jianyong Wu, Jiyoung Lee

**Affiliations:** 1 Environmental Sciences Graduate Program, The Ohio State University, Columbus, OH 43210, USA; byrd.350@buckeyemail.osu.edu; 2 Division of Environmental Health Sciences, College of Public Health, The Ohio State University, Columbus, OH 43210, USA; wu.6255@osu.edu; 3 Department of Food Science & Technology, The Ohio State University, Columbus, OH 43210, USA

**Keywords:** harmful algal blooms, unmanned aerial vehicles, cyanotoxins, public health, remote sensing, chlorophyll-a, phycocyanin, multispectral imaging, hyperspectral imaging

## Abstract

Harmful algal blooms (HABs) are an escalating global concern due to their increasing frequency, duration, intensity, and geographic spread. These events threaten public health by contaminating drinking water sources, recreational areas, and food production systems with cyanotoxins. Effective monitoring is critical but remains limited by the spatial and temporal variability of blooms. Unmanned aerial vehicles (UAVs) have recently emerged as a flexible, high-resolution tool for HAB monitoring that can complement satellite and in situ methods. This review synthesizes recent applications of UAVs in HAB detection, mapping, and sampling, with a focus on how these approaches can support public health interventions. Key UAV platforms, sensor types, and data processing workflows are summarized, along with considerations related to flight regulations. Studies linking UAV data to indicators like chlorophyll-a and phycocyanin are discussed, highlighting their relevance for early warning systems and water treatment responses. Finally, the review identifies persistent challenges—including validation, regulatory gaps, and integration with health risk frameworks—and provides recommendations to advance UAV-based monitoring. These insights support the continued development of UAV systems as part of comprehensive strategies to mitigate HAB-related health risks.

## 1. Introduction

Cyanobacterial blooms occur in both coastal and inland water bodies worldwide [1]. As primary producers, cyanobacteria play a vital role in aquatic food webs when balanced within ecosystems [2]. However, they can proliferate rapidly in response to environmental conditions such as elevated nutrient concentrations, wind speeds, water temperatures, and light intensity [3]. This rapid growth negatively impacts aquatic ecosystem health, local economies, and human well-being. Many blooms produce hazardous bioactive compounds known as cyanotoxins, which fall into five main categories: hepatotoxins, neurotoxins, dermatotoxins, cytotoxins, and irritant toxins [4]. These toxins pose significant health risks, leading to both acute and chronic effects, including neurological, liver, reproductive, and digestive disorders. Reported symptoms of cyanotoxin exposure include diarrhea, vomiting, fever, allergic reactions, gastrointestinal distress, paralysis, organ damage, and tumor promotion. Common exposure pathways include inhalation, dermal contact, and ingestion of contaminated water or food [4], particularly during drinking, swimming, or recreational activities such as boating, fishing, or water skiing [5].

Recent studies have shown an increase in the frequency, duration, and geographic extent of harmful algal blooms (HABs) worldwide, driven by urbanization, global warming, and eutrophication [6]. As a result, the risk of human exposure to cyanotoxins and the burden on water utilities have grown. Monitoring HABs and their associated toxins is essential for developing early warning systems to protect public health. Currently, four main monitoring strategies are used: (1) ground sampling, (2) on-site sensor networks, (3) satellite remote sensing, and (4) airborne remote sensing [7]. Ground sampling, though widely used, is time-consuming and resource-intensive, requiring transportation, sample processing, and lab analysis [3]. These limitations result in low spatial and temporal coverage, which is inadequate for capturing HAB dynamics [1]. On-site sensor networks, such as monitoring buoys, improve sampling frequency but only provide data near the station and do not represent entire water bodies.

Satellite remote sensing uses multispectral and hyperspectral sensors to detect water quality indicators like chlorophyll-a (chl-a), phycocyanin (PC), and colored dissolved organic matter (CDOM). While chl-a and PC are photosynthetic pigments, they also influence water color and can be optically measured. Satellite systems offer broad spatial coverage but have two main limitations. First, temporal resolution is restricted by revisit intervals and atmospheric interference such as cloud cover, shadows, and snow—especially in high-latitude areas [8]. For instance, the Moderate Resolution Imaging Spectroradiometer (MODIS) and Medium Resolution Imaging Spectrometer (MERIS) revisit sites every 16 and 35 days, respectively [2], leaving large data gaps [9]. Second, spatial resolution is often too coarse to monitor smaller water bodies; for example, MODIS imagery may have a resolution of 250–1000 m. Manned aircraft can acquire high-resolution data and avoid cloud interference, but they are costly, pose risks in poor weather, and can disturb wildlife.

Unmanned aerial vehicles (UAVs) have recently emerged as a low-cost, safe, and flexible airborne remote sensing option [10]. UAVs can be deployed on demand, fly below cloud cover, and capture centimeter-scale imagery. They also support a variety of payloads, including red–green–blue (RGB) cameras, thermal sensors, light detection and ranging (LiDAR), multispectral and hyperspectral sensors, and water sampling devices. However, UAV-based monitoring faces challenges such as image processing difficulties, limited spatial coverage, and a lack of standardized methods [8]. Still, their use in HAB monitoring is growing. Since the last major review by Wu et al. in 2019, UAV technologies and regulations have evolved rapidly [2]. The purpose of this review is to synthesize UAV-based remote sensing and sampling applications from 2017 to the present, identify best practices, and evaluate their feasibility for supporting public health interventions. We summarize hardware, software, and analytical techniques, discuss current limitations and regulatory considerations, and provide recommendations for future research. To our knowledge, this is the first review to examine UAV applications in HAB monitoring through a public health lens.

This review includes 27 peer-reviewed journal articles published from 2017 to 2024. The starting year of 2017 was selected to build upon the most recent comprehensive review, which only included studies up to that point. Articles were identified primarily through Google Scholar using combinations of relevant search terms, including “HAB,” “drone,” “public health,” “algae blooms,” “UAV,” “red tide,” “UAV sampling,” and “drone sampling.” While Google Scholar was the main search engine used, its broad indexing was supplemented by manual screening to exclude unrelated results. Future reviews may benefit from parallel searches using structured databases such as Web of Science or Scopus. Only English-language, peer-reviewed journal articles were included; dissertations, theses, and conference proceedings were excluded. Papers were selected if UAVs were used as a meaningful component of the research, if flight details were provided, and if the monitoring approaches could be applied to blooms of public health concern. The type of UAV platform was not a basis for inclusion or exclusion. Studies that focused solely on unmanned surface vehicles (USVs) were excluded as they fall outside the scope of this review. Collected papers spanned diverse water body types, methodologies, and geographic locations, and were thoroughly reviewed to produce a current and comprehensive synthesis of the field.

## 2. Applications of UAVs for Protecting Public Health

UAVs have been widely applied in HAB monitoring due to their flexibility, relatively low cost, and ease of data collection. The summary of 27 peer-reviewed studies from 2017 to the present, shown in Supplementary Information (Table S1) [3,7,8,11,12,13,14,15,16,17,18,19,20,21,22,23,24,25,26,27,28,29,30,31,32,33], is organized chronologically, along with each study’s potential contribution to public health policy.

**Figure 1:**
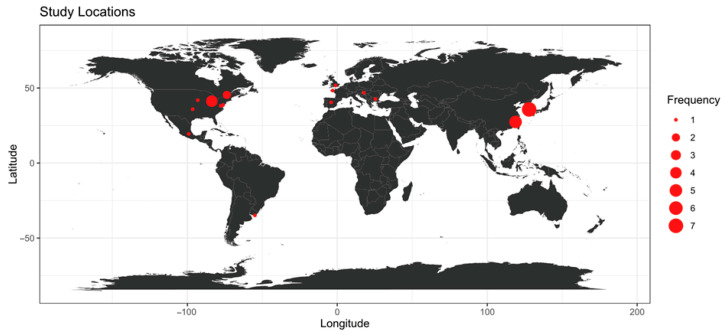
Geographical distribution of studies utilizing UAVs for HAB research.

**Figure 2:**
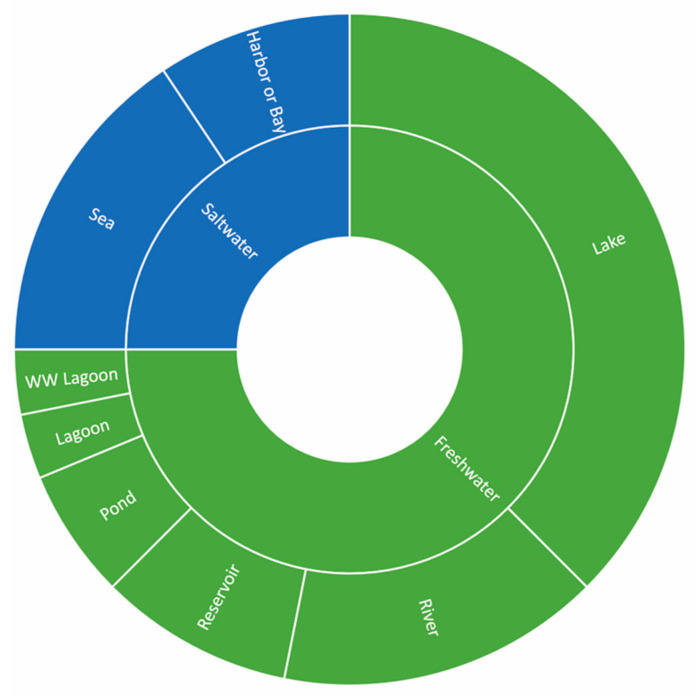
Types of water bodies assessed across reviewed studies. “WW Lagoon” refers to wastewater lagoons. Studies involving multiple water body types are represented in each applicable category. Classifications are presented as reported in the original publications and were not altered.

These studies span a global range, with South Korea appearing most frequently. The geographic distribution of studies is shown in Figure 1, while Figure 2 illustrates the diversity of water bodies assessed, including both saltwater and freshwater systems.

**Figure 3:**
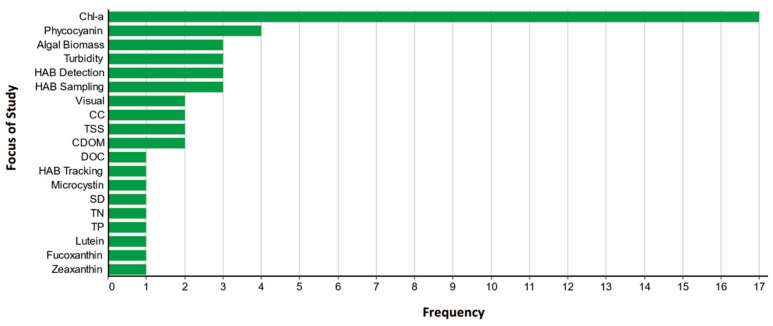
Research focus areas across all reviewed studies. If a study addressed multiple topics, each relevant focus area was included. Abbreviations: CDOM = colored dissolved organic matter; CC = cyanobacteria concentration; TSS = total suspended solids; DOC = dissolved organic carbon; SD = Secchi disk depth; TN = total nitrogen; TP = total phosphorus.

The variety of objectives across these studies reflects the broad applicability of UAVs for HAB monitoring. Many focused on quantifying photosynthetic pigments associated with cyanobacteria, though the target pigment varied by study. Commonly assessed pigments included chl-a, PC, CDOM, total suspended solids (TSS), lutein, fucoxanthin, and zeaxanthin [33]. Several studies integrated UAV-derived imagery with in situ water quality data to develop predictive models [29]. A recurring theme was the need for standardized image processing workflows; Windle et al., for example, compared four different approaches for removing sun glint to improve UAV-derived chl-a estimates [28]. Some studies paired UAV data with satellite imagery to enable multi-scale monitoring frameworks [8,9], leveraging satellite platforms for regional-scale insights and UAVs for fine-scale, local detail. Other research focused on UAV-enabled environmental sampling, such as water and aerosol collection, with significant reductions in sample collection time reported [31,32]. UAVs were also used to identify optimal sampling sites or to validate novel HAB detection techniques [20]. One study demonstrated real-time UAV-based monitoring to detect bloom position and movement near a desalination plant, enabling improved operational decisions [16]. Overall, UAVs were applied across a range of use cases, from imagery-based pigment estimation to direct environmental sampling. Figure 3 summarizes the various functions UAVs served in these studies, with chl-a estimation being the most common application.

### 2.1. Studies Focused on Chlorophyll-A Estimation

Chl-a estimation was the most common UAV application in HAB research, despite ongoing challenges in image processing and a lack of standardized workflows. Of the 27 studies reviewed, 16 included chl-a analysis, with seven focused exclusively on it. To address the need for standardized processing, several studies introduced novel methods for deriving chl-a concentrations from corrected reflectance measurements. Shang et al. developed a method to retrieve remote sensing reflectance (Rrs) in nearshore environments using UAVs with spectroradiometers [12]. Their approach reduced error in derived Rrs by correcting for environmental interference (e.g., atmospheric scattering, whitecaps, cloud movement), which is essential for ensuring that water resource managers can rely on UAV-derived pigment data when determining whether to close intakes during bloom events. A two-step quality control process was implemented before deriving Rrs, involving wavelet transform outlier removal followed by land masking [34]. Cheng et al. introduced a radiometric correction technique using RGB digital cameras, converting raw digital number (DN) values to corrected DN via a calibration panel [22]. These values were then used to derive chl-a concentrations providing a low-cost option for communities that lack access to advanced sensors but still require accurate data to issue public health advisories. Shang et al. applied an empirical algorithm to convert UAV-derived Rrs into chl-a estimates, with <20% error compared to in situ measurements—substantially lower than the 136% error reported for MODIS-derived estimates [12]. Cheng et al. evaluated six band ratio combinations to model chl-a and found the red/blue ratio to be optimal for RGB cameras, though predictive performance declined at concentrations >15–20 μg/L [22]. While this limits fine-scale quantification during severe blooms, the approach can still function as a categorical warning tool, distinguishing between risk and no-risk conditions for issuing public health advisories.

Three studies estimated chl-a concentrations using UAVs equipped with multispectral sensors. Baek et al. developed a multiple linear regression model to relate Rrs at each wavelength to in situ chl-a measurements [17]. Applying the resulting algorithm to UAV imagery yielded strong agreement with ground-truth data, with a correlation coefficient of 0.94 and a root mean square error (RMSE) of ~0.8 μg L^−1^. Choo et al. used a linear regression model to link chl-a concentrations with the normalized difference vegetation index (NDVI), reporting an R^2^ of 0.70, indicating good correlation between NDVI and in situ chl-a values [14]. Such correlations enable UAV imagery to be used as a screening tool for health agencies, particularly when laboratory turnaround times might delay the issuance of recreational advisories. Tóth et al. used three camera outputs—light level, irradiance, and reflectance—to estimate chl-a concentrations based on blue/green and near-infrared (NIR)/red band ratios [35]. First-order equations were developed between band ratios and lab-derived chl-a values. A scaling multiplier was applied to match UAV outputs with laboratory results, and deviations were assessed across five distinct water bodies with varying physical characteristics. The strongest correlations were observed using blue/green light level ratios. Notably, the study reported that UAV sampling reduced the time required for chl-a estimation by approximately 50% compared to lab-based analysis, shortening the window between bloom detection and public health warnings.

Optical sensors for estimating chl-a are increasingly used in water resource management but often require model reparameterization for each water body due to variability in local conditions. This limits their immediate utility for public health decision-making, since models tuned to one lake may underperform in another where advisories are needed most. To address this limitation, El-Alem et al. developed a UAV-based model for chl-a estimation that could be applied to Sentinel-2 satellite imagery without reparameterization [8]. An ensemble-based system (EBS) algorithm was trained using in situ samples from three lakes with varying trophic statuses, providing a wide range of chl-a values for calibration. Model performance was assessed at two scales: the local scale used leave-one-out cross-validation within the training lakes, while the regional scale was evaluated using a blind dataset of 94 samples from 89 independent lakes not used during training. UAV imagery was preprocessed to align with Sentinel-2 spatial and spectral characteristics, including: (1) geometric and radiometric correction, (2) spatial upscaling, (3) Sentinel-2 band simulation, and (4) reflectance computation. Cillero Castro et al. also developed a multi-scale, multi-sensor chl-a monitoring framework combining multispectral satellite imagery, UAVs, and in situ data [9]. Empirical models were built by linking in situ chl-a concentrations to Rrs across 16 band combinations. Model performance in both studies was evaluated using root mean square error (RMSE) and bias. Additionally, El-Alem et al. [8] used the Nash criterion and relative error, while Cillero Castro et al. employed normalized RMSE (NRMSE) and mean absolute percentage error (MAPE) [9]. Although both models performed well, accuracy declined for high chl-a concentrations and in turbid waters. From a public health perspective, UAV–satellite frameworks are best suited for early warning and tracking moderate blooms, but severe events—when toxin exposure risks peak—still demand additional validation to avoid underestimating the need for advisories. The authors emphasized the need for large, diverse datasets and recommended further research on algorithms calibrated across broader chl-a ranges.

### 2.2. Studies Focused on Phycocyanin Estimation

Although PC is a more specific indicator of cyanobacterial presence than chl-a, fewer studies have focused on its estimation due to sensor limitations [35]. Most UAV-compatible sensors lack the spectral resolution needed for PC detection, which typically requires more expensive and heavier hyperspectral sensors. All studies estimating PC also estimated chl-a concentrations [11,21,29]. One study additionally measured other pigments, including lutein, fucoxanthin, and zeaxanthin [33]. Aguirre-Gómez et al. estimated PC concentrations using a UAV equipped with an RGB sensor [11]. The method leveraged PC’s absorption peak at 619 nm, in conjunction with in situ hyperspectral radiometer measurements and cyanobacterial lab analysis. Mean value maps of the PC absorption peak were generated using the inverse distance weighting method, which interpolates non-sampled locations based on nearby measured values. This type of mapping provides a complete spatial overview of bloom intensity that could help local agencies determine where targeted sampling is needed.

Three studies estimated PC concentrations using UAVs equipped with hyperspectral sensors. Kwon et al. investigated how diel vertical migration of cyanobacteria affects UAV-derived PC estimates by generating vertical pigment concentration profiles at the surface and three subsurface depths using a portable Rrs sensor and UAV imagery [21]. Bio-optical algorithms were developed and compared, with the best-performing model—based on a two-band ratio (709 nm/620 nm)—used to estimate PC concentrations and generate distribution maps. By capturing pigment signals linked to different depths in the water column, this approach offers insight into subsurface cyanobacterial accumulation that is often invisible to surface-only monitoring but still poses risks to drinking water intakes. Pyo et al. and Hong et al. applied deep learning models to UAV hyperspectral data for PC estimation [29,31]. Like Kwon et al. [21], Hong et al. aimed to capture vertical PC distribution [29]. Four deep neural network models were trained using UAV and meteorological inputs to produce vertical water quality profiles. After hyperparameter optimization, the models were applied to UAV imagery to generate spatial PC maps. Pyo et al. used a one-dimensional convolutional neural network to estimate PC and other pigments (chl-a, lutein, fucoxanthin, zeaxanthin) [33]. The trained model was applied to UAV reflectance maps to create pigment absorption coefficient maps and corresponding spatial distribution outputs. All three studies reported model performance using R^2^ and root mean square error (RMSE), with Pyo et al. also using mean absolute percentage error (MAPE), mean absolute error (MAE), and the Willmott Agreement Index (WAI) [29]. Collectively, these studies suggest that hyperspectral UAVs, when paired with advanced modeling, can help identify bloom “hot spots” and stratification patterns most relevant to human exposure, guiding water treatment actions and reducing HAB-related health risks.

### 2.3. Non-Pigment UAV Applications: Toxins, Biomass, and Sampling

The remaining 16 studies used UAVs for HAB monitoring by targeting parameters beyond chl-a and PC, though some still included these pigments. These studies fell into five broad categories: microcystin (MC) evaluation, HAB sampling, HAB detection, algal biomass/concentration estimation, and assessment of various water quality components. One study attempted to predict MC concentration distributions using a UAV equipped with a multispectral sensor. In situ and remotely sensed data were analyzed across seven vulnerable lakes in Iowa, USA, and correlations between chl-a and MC concentrations were evaluated. Results showed that these relationships varied widely between lakes, with MC concentrations predicted within a 33% margin of error. Given the public health risks posed by MCs in drinking water, this level of uncertainty is inadequate for decision-making, underscoring that UAV-based MC estimation is not yet reliable enough to support advisories without extensive ground validation [27].

While most studies used UAVs for remote sensing, three focused on HAB sample collection. One study developed an early detection system using two UAVs—one for HAB detection and another equipped with a custom sampling device capable of collecting seawater at depths of 1, 3, and 5 m during a single flight [19]. Haulon et al. deployed a custom 3D-printed DrOne Water Sampling System (DOWSE) to collect 180 surface water samples across three water bodies [32]. The UAV simultaneously captured photographs and GPS coordinates, improving sampling efficiency to approximately 12 min per 10 samples. Such rapid, spatially explicit sampling could strengthen the timeliness of advisories issued to recreational users. Bilyen et al. designed and deployed the Airborne DROne Particle-monitoring System (AirDROPS) to collect and characterize airborne particles over HABs. Particle counts from AirDROPS closely matched those from a commercial particle counter [31]. By addressing the potential for airborne cyanotoxin exposure, this approach extends UAV monitoring beyond water to inhalation pathways.

Four studies used UAVs to detect cyanobacterial blooms. Stoyneva-Gärtner et al. conducted aerial surveys to visually identify HABs [20]. One study used UAV imagery to guide water sampling site selection, while another combined acoustic Doppler current profiler (ADCP) data with UAV flights to verify predicted HAB-prone areas along a river [24]. Lyu et al. developed a framework for optimizing UAV flight parameters and image quality, offering guidance for effective HAB monitoring system design [7]. Kim et al. implemented a real-time HAB detection system near desalination plants using UAV imagery and a color detection algorithm based on the Hue, Saturation, Value (HSV) model [16]. Once a bloom was detected, its direction and velocity were calculated, demonstrating how UAV systems can directly protect public health by providing real-time information to guide operational decisions around water intakes. The system achieved over 80% accuracy in HAB extraction, though additional refinement was needed for motion tracking.

Four studies evaluated algal biomass or concentration, with two focusing on biomass and two on cyanobacteria concentration (CC). One study identified green algae using a UAV with an RGB camera and developed a biomass estimation model based on Sentinel-2A (S2A) satellite imagery [13]. A second study estimated red algae biomass using UAV-acquired multispectral imagery and regression models based on four vegetation indices [30]. Barruffa et al. and Becker et al. estimated CC using multispectral sensors and spectroradiometers, respectively [3,15]. Barrufa et al. developed a novel image processing workflow and regression models to correlate spectral bands and ratios with water quality parameters, demonstrating that a 4-band multispectral camera could distinguish cyanobacteria from other photosynthetic organisms [3]. This level of specificity is important because cyanobacteria pose the greatest public health risks through toxin production. Becker et al. deployed two custom UAVs with spectroradiometers to estimate CC. After image processing, four algorithms were applied to generate cyanobacteria index (CI) products, which showed strong agreement with ground-based reflectance values [15].

The remaining four studies assessed chl-a alongside additional water quality parameters. One study evaluated methods for removing light surface reflectance (LSR) in UAV-based hyperspectral imagery to improve estimation accuracy for chl-a and TSS. Their results identified blue, red, red-edge, and near-infrared (NIR) bands as most important for TSS prediction [28]. Arango et al. assessed total phosphorus (TP), total nitrogen (TN), and Secchi disk depth (SD) using UAV imagery and developed single- and multi-variable linear regression models [18]. McEliece et al. estimated the spatial distribution of chl-a and turbidity in a marine environment using a multispectral UAV platform [23]. They created and validated calibration functions, then tested them on an independent dataset. Luo et al. examined eutrophication dynamics in a desert lake and estimated CDOM using a UAV equipped with an RGB camera [26]. CDOM absorption coefficients served as concentration indicators, with blue and green band ratios yielding the best estimation results. Across all four studies, once optimal band ratios were identified and algorithms validated, spatial distribution maps were generated. When interpreted alongside HAB indicators such as chl-a, these maps can reveal interactions among water quality parameters that shape bloom dynamics and inform assessments of public health risk.

## 3. UAV Hardware and Software for HAB Monitoring

### 3.1. Overview of UAV Platforms

Unmanned aerial vehicles (UAVs) come in various designs, each with strengths and limitations that must be matched to the specific research goals [10]. The three main commercial UAV categories are fixed-wing aircraft, rotorcraft (multirotor), and fixed-wing vertical takeoff and landing systems (VTOL) [36], with less common systems like blimps also occasionally used. In this review, rotorcraft were the most widely used (93%), followed by fixed-wing aircraft (7%); no VTOL systems were reported. Blimps were only used in one study and are grouped here with rotorcraft due to their hovering capabilities [16]. Rotorcraft were typically chosen due to their lower cost, ease of deployment, and suitability for confined launch areas such as boats or vegetated shorelines. Fixed-wing aircraft offer longer flight times and greater range but lack hovering capabilities. VTOLs combine vertical takeoff with longer flight endurance, but are more expensive and less commercially available, with limited supporting software [1]. UAV performance is also influenced by environmental factors such as wind speed, temperature, and humidity, which affect battery life and flight stability. Table 1 summarizes the advantages and disadvantages of each UAV type.

**Figure 4:**
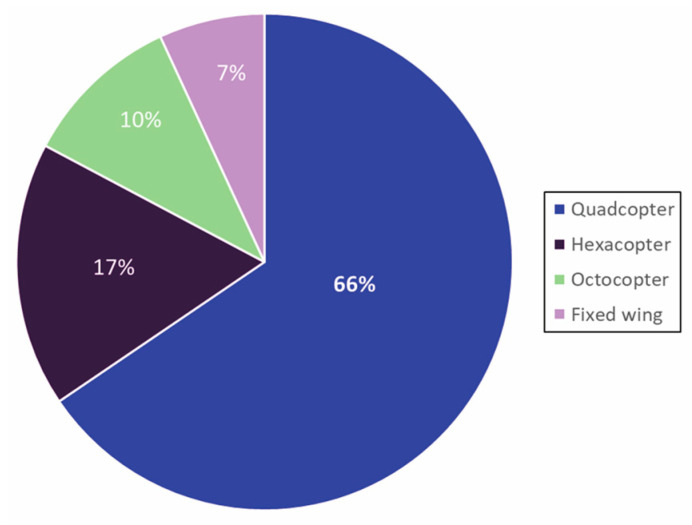
Distribution of UAV types used across reviewed studies. Percentages indicate frequency of use. Studies employing multiple UAV types are represented in each applicable category.

Among rotorcrafts, 66% of studies used quadcopters, 17% hexacopters, and 10% octocopters (Figure 4), likely due to cost differences and payload needs. Many studies requiring low altitude hovering or launch from small areas favored quadcopters. Although rotorcrafts dominated this review, no conclusive evidence suggests they outperform other platforms universally. Platform selection should be tailored to specific monitoring needs, costs, and operational constraints. To date, no study has directly compared fixed-wing, rotorcraft, and VTOL outputs [37,38].

Several studies modified commercially available UAVs or built custom platforms to meet unique research needs, such as 3D-printed mounts and landing gear extensions to support custom sensors [28,35]. Becker et al. constructed a waterproof UAV with a Pixhawk PX4 flight controller and hyperspectral camera for under $2000 (excluding sensor cost) [15]. One study reported the AirDROPS system, integrating a particle impinger, optical particle counter, and environmental sensors into a UAV for airborne HAB particle monitoring, mounted to reduce propeller downwash [31]. Other researchers customized UAVs for water sampling. Retrofitted a quadcopter was developed with a 3D-printed DOWSE (DrOne Water Sampling SystEm) device and tether line, collecting surface samples while capturing GPS coordinates and imagery of each site [32]. Kimura et al. developed a pulley-based water sampler mounted to a DJI-based quadcopter, enabling remote collection of samples at 1, 3, and 5 m depth via pressure-triggered valves [19]. Collected samples were analyzed microscopically, and real-time alerts were sent to public health officials, demonstrating UAVs’ potential for rapid decision support and near immediate risk communication. While rotorcraft were the most commonly used, platform choice remains context dependent. Factors such as payload, maneuverability, launch conditions, and mission duration should guide UAV selection, and as VTOLs become more affordable and software expands, their use in HAB monitoring may grow.

### 3.2. Sensor and Payload Configurations

**Figure 5:**
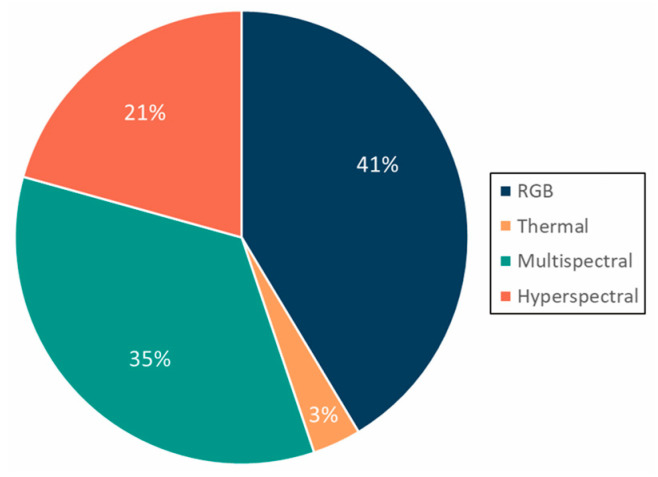
Sensor types used across studies. Percentages reflect the frequency of use, with studies employing multiple sensors counted in each relevant category.

UAVs, especially rotorcrafts, can be equipped with a range of attachments, most commonly sensors and sampling devices. Sampling attachments included water and aerosol samplers, which were detailed in the previous section. This section focuses on the four sensor types used in the reviewed studies: RGB, multispectral, hyperspectral, and thermal. RGB cameras were the most frequently used (41%), likely due to their affordability and widespread inclusion on commercial UAVs. Multispectral sensors followed (35%), then hyperspectral (21%), and thermal (3%) (Figure 5).

Each sensor type offers trade-offs. RGB cameras, while cost-effective, are limited to three bands (red, green, blue), restricting analytical applications and providing only coarse indicators of bloom presence. This lower specificity reduces their reliability for informing public health actions, where false positives could lead to unnecessary advisories. Thermal cameras, used in only one study [25], offer utility for analyzing algal warming events [39], especially when paired with multispectral data. Multispectral sensors extend data collection beyond visible light by splitting electromagnetic waves into narrower bands through photoelectric detection [40]. This allows extraction of spectral features to identify targets like cyanobacteria among other photosynthetic organisms [3]. Though more expensive than RGB, multispectral sensors offer richer spectral resolution at a lower cost and payload burden than hyperspectral sensors. Hyperspectral sensors, though costly and heavier, capture hundreds of bands, enabling detailed pigment analysis and facilitating the simulation of satellite bands through resampling [15]. These are particularly valuable when high spectral precision is needed, such as estimating HAB pigment composition or testing remote sensing algorithms. Sensor selection should align with research goals. In the context of public health, RGB sensors provide low-cost tools for broad screening, multispectral sensors enable more quantitative risk assessment of bloom indicators such as chl-a and PC, and hyperspectral sensors—despite higher cost and payload demands—offer the greatest potential for identifying toxin-producing species when precise information is needed for decision-making [38].

### 3.3. Configurations and Corrections for Reflectance Retrieval

Accurate retrieval of Rrs from UAVs is essential for generating reliable data needed to support public health decisions, and requires measurement of downwelling irradiance, separation of water-leaving radiance, and appropriate control of sensor viewing geometry. Across the reviewed studies, these elements were handled inconsistently and differently based on sensor type. Hyperspectral systems most frequently implemented complete optical configurations. Several studies paired spectroradiometers or cosine receptors with UAV sensors to measure downwelling irradiance and water-leaving radiance, while also orienting sensors at Mobley-recommended geometries (~40° off-nadir, 135° azimuth) to reduce specular reflection [12,15,21,29,33]. Reflectance correction methods included Dark Object Subtraction [1], sky radiance subtraction using Mobley’s q-factor [21], O_2_-band sun glint removal [12], white and dark reference panels [29], and Cosine of the Solar Zenith Angle (COST) and modified COST atmospheric models [8]. Advanced filtering such as Savitzky–Golay smoothing and Wiener2 noise reduction was also applied to hyperspectral imagery [29]. These approaches yielded strong agreement with in situ spectroradiometers, enabling accurate retrievals of chl-a, PC, and accessory pigments.

Multispectral platforms typically incorporated downwelling light sensors and calibration reflectance panels to normalize illumination [14,17,18,22,23,27]. However, they rarely collected direct water-leaving radiance. These approaches focused on radiometric normalization and geometric processing were performed using commercially available orthomosaic software [18,27]. Additional corrections involved vignetting and lens distortion adjustments [27], and empirical reflectance scaling [23]. Several studies also attempted to mitigate sunglint, either through flight timing (solar zenith < 60°) [22], NIR subtraction [17], or Hedley-style deglinting [3]. These approaches improved consistency but were less robust across waterbodies, and predictive performance declined at high pigment concentrations.

RGB-only applications largely lacked irradiance or radiance sensors, relying instead on minimal corrections such as gimbal stabilization, orthorectification, or glare filters [7,13,16,20,26]. Some attempted algorithmic workarounds—for instance, HSV color space conversion to adjust for brightness variability [16] or basic atmospheric corrections via ACOLITE for Sentinel-2 integration [13]—but without radiometric calibration, RGB systems remained prone to false positives caused by surface reflection and limited spectral specificity. In summary, hyperspectral UAV studies implemented the most comprehensive optical configurations, while multispectral platforms generally applied partial corrections and RGB systems were the least rigorous. These differences underscore the need for standardized workflows to ensure consistent and reliable reflectance retrieval across UAV-based HAB monitoring efforts.

### 3.4. Image Processing Software and Programming Tools

**Figure 6:**
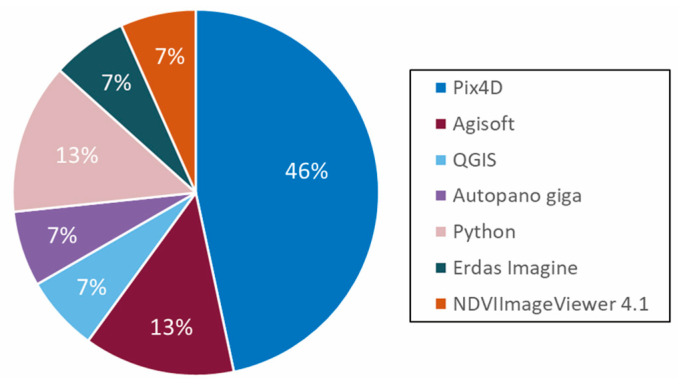
Software and programming languages used across studies. Percentages reflect frequency of use. Studies using multiple tools were counted for each. Studies that did not specify software or generate spatial products were excluded.

General steps for UAV image processing include reflectance calibration, photo alignment, radiometric calibration, atmospheric correction, and post-processing [41]. However, no standard pipeline currently exists, and methodologies vary widely across studies. The tools used often depend on the sensor type: while RGB, thermal, and multispectral imagery can generally be processed by the same software, hyperspectral data requires specialized tools due to its volume and format. SpectralView was the only software used for hyperspectral image processing in the reviewed studies. In contrast, RGB, thermal, and multispectral imagery were processed with a variety of platforms. Of the reviewed studies, 46% used Pix4D, 13% Agisoft, 13% Python, and 7% each used one of four other tools (Figure 6).

Several studies used software and programming languages in tandem. For instance, Python was used to geo-reference, rotate, and convert images to reflectance, followed by Erdas Imagine to mosaic and apply band math [27]. Although no HAB-focused studies directly compared software outputs, other work suggests differences in performance. One study reports that Pix4D aligned fewer images than Agisoft but had lower XY error, while Agisoft had lower Z error [42]. It was also noted Agisoft’s popularity in surface reconstruction workflows [43]. Given the challenges of mapping HABs—such as water movement and homogeneity—future studies may benefit from software comparisons tailored to aquatic environments.

## 4. Measurement and Modeling of Cyanobacteria-Associated Metrics

### 4.1. Spectral Indices for Cyanobacteria Quantification

Various spectral band combinations—expressed as indices, band ratios, or algorithms—were used for HAB detection and monitoring [9]. Table 2 summarizes the pre-defined indices and their band math. Studies that did not use or specify indices, or developed custom band ratios or algorithms, are excluded from the table and discussed in Section 4.3.

Of the seven studies using pre-defined indices, five applied multiple methods to identify optimal performance. NDVI was solely applied for image correction to differentiate water, emergent vegetation, and submerged plants [3]. NDVI was also used with a strong correlation with chl-a (R^2^ = 0.70) [14]. One study tested 16 indices; B3B1, GB1, and G/R performed best for chl-a retrieval, with B3B1 showing the strongest correlation—suggesting blue-green band combinations are effective for distinguishing variable chl-a concentrations [9]. For public health officials, such correlations are critical because they determine whether UAV-derived maps can reliably flag bloom intensities that approach thresholds of concern for cyanotoxin production. It was shown that NGRDI was the most accurate among four tested indices, while NGBDI could distinguish algae from synthetic objects like blue ropes, albeit with some misclassification [13]. The two indices were combined for improved algal detection, highlighting the importance of indices selection in avoiding false positives that could either overstate or understate bloom-related health risks. It was noted that DVI, RVI, NDVI, and NDRE all correlated strongly with algal biomass, reinforcing their potential as early warning indicators when integrated into advisory frameworks [30]. However, one study found poor correlation between all eight indices tested and measured chl-a, with SHI achieving the highest R^2^ (0.18) [27]. The authors attributed this to suboptimal weather conditions during UAV data collection, emphasizing the importance of clear weather and a broad range of HAB concentrations for reliable model calibration.

### 4.2. Ground-Truthing and Accuracy Assessment Techniques

Since UAV-based imagery processing is not yet standardized, all studies included some form of validation against traditional methods [3]. Most collected water samples immediately before or after UAV flights, with GPS coordinates recorded for each site. Laboratory analysis of chl-a was typically performed using spectrophotometers or fluorometers, while some studies also used microscopy to identify algal species. To validate reflectance values, several studies employed handheld spectroradiometers to measure surface reflectance, allowing comparison with post-processed UAV imagery. These comparisons assessed the accuracy and effectiveness of the image processing workflow. Validation is particularly important for public health because it reveals the limitations of UAV imagery and ensures that any uncertain outputs are interpreted cautiously, supporting better-informed decisions about bloom risk. In summary, incorporating ground-truth validation, whether through lab analysis or reflectance comparison—is essential in UAV-based HAB monitoring.

### 4.3. Computational Models for HAB Forecasting and Analysis

Advancements in UAV platforms and sensors have enabled the development of diverse HAB detection algorithms, generally falling into three categories: empirical, semi-empirical (ratio-based), and advanced statistical approaches such as spectral derivative models [8,44]. Several studies demonstrated successful application of existing algorithms for chl-a estimation. For example, HSV-based color detection algorithm was used to extract HABs from UAV imagery with over 80% efficiency [16]—capable of tracking consistent color despite shifts in brightness or position. Shang et al. applied an empirical model based on wavelength-shift and fluorescence line height algorithms [12], supporting UAVs as useful tools during HAB events [45,46]. Most studies employed empirical or semi-empirical models, while a few incorporated more advanced statistical or machine learning methods. Quantitative approaches were generally grouped into two main types: linear regression and machine learning models.

Linear regression was the most widely used modeling approach due to its simplicity. B/G band ratio from UAV imagery was used to estimate CDOM absorption coefficients across three crop growth stages, showing strong correlations with fDOM probe data (r = 0.85, 0.86, 0.71) [26]. These stages corresponded with irrigation-driven fluctuations in lake levels, as nearby croplands relied on groundwater and lake water for irrigation. During early and middle growth stages, high water demand caused a sharp drop in lake levels and potentially concentrated CDOM, while the reduced demand in the later stage allowed groundwater recharge. Consequently, tracking crop growth stages helped contextualize changes in eutrophication levels. Similarly, it was found the B/G ratio using light-level data outperformed NIR/R ratios for chl-a estimation [35] and 450 surface chl-a measurements were used to compare linear, power, and exponential models, identifying R/B as the best input for a linear model (R^2^ = 0.84; RMSE = 3.17 µg/L), though accuracy declined above 20 µg/L [22]. Using hyperspectral data, semi-empirical models were developed for PC and chl-a based on linear regressions of band ratios (709/620 nm for PC and 709/665 nm for chl-a), achieving R^2^ values up to 0.82 [21]. Orthogonal regression was applied to calibrate a chl-a model using 448 and 550 nm wavelengths, explaining 78% of the variance [23].

Multiple linear regression (MLR) models were frequently used to improve predictions by incorporating multiple spectral bands. Several band ratios, G, RE, G/RE, R/NIR, RE/NIR, R/NIR2, R^2^, and RE/NIR2, were evaluated [3], accounting for limnological influences on chl-a estimation [47]. Their best MLR model achieved R^2^ = 0.79 for chl-a and 0.77 for colored compounds (CC). Another study used both single and MLR models to estimate TSS, chl-a, SDD, TP, and TN, with the best chl-a model (R^2^ = 0.846) using green and red bands [18]. Another study also applied MLRusing blue, green, and red bands for chl-a estimation, red edge for suspended matter differentiation, and NIR for sun glint correction, achieving a high R^2^ = 0.942 between UAV-derived and in situ chl-a values [17]. Windle et al. reported an MLR model for chl-a with R^2^ = 0.43 and RRMSE = 37% [28], comparable to other studies [48,49], which reported errors of 9.2 µg/L, 37%, and 51.9%, respectively.

Advanced statistical methods such as ensemble-based systems (EBS) and deep learning were also applied for HAB estimation. El-Alem et al. developed a chl-a model using an EBS applied to both UAV and satellite imagery [8]. To enhance accuracy, separate models were created for oligotrophic, mesotrophic, and eutrophic waterbodies. A similar strategy, grouping data by five water level categories to improve regression model accuracy, was used [25]. The EBS achieved strong performance at both local and regional scales, with R^2^ = 0.94, Nash = 0.94, RMSE = 5.6 μg/L (local), and R^2^ = 0.85, Nash = 0.79, RMSE = 2.4 μg/L (regional). The Nash coefficient (Nash–Sutcliffe efficiency) is a normalized statistic that compares the predictive power of a model to the mean of observed values; values closer to 1 indicate better performance. However, the model struggled in highly turbid waters. Deep learning approaches also showed promise. One study used a 1D convolutional neural network (CNN) to estimate chl-a, phycocyanin (PC), lutein, fucoxanthin, and zeaxanthin using UAV-derived reflectance and absorption data [33]. The CNN outperformed bio-optical algorithms, with the highest R^2^ = 0.87 for chl-a (vs. R^2^ = 0.66 for the algorithm). It was also applied four CNN architectures (ResNet-18, ResNet-101, GoogleNet, Inception v3) to UAV hyperspectral data for estimating vertical HAB distributions [29]. ResNet-18 achieved the best performance (R^2^ = 0.7). Although not applied directly to UAV imagery, [19] trained a CNN on UAV-collected water samples to classify five plankton species, reaching 99.5% accuracy—enabling rapid detection of hazardous taxa. From a public health standpoint, the challenge now lies in translating these computational advances into standardized, user-friendly tools so that water managers and health agencies can apply UAV-derived forecasts without requiring advanced statistical or programming expertise.

## 5. Discussion

### 5.1. Practical and Environmental Challenges in UAV-Based HAB Monitoring

Researchers frequently encountered challenges such as adverse weather (cloud cover, wind, rain), difficulty stitching images over water, and interference in spectral data [27]. These conditions negatively impacted model development by introducing noise or altering reflectance values [20,50]. Minimizing the lag between UAV and in situ data collection is crucial, as solar illumination can significantly alter water reflectance, causing specular reflection or shadows that reduce accuracy [14,15,51]. Flights during mid-morning or mid-afternoon were recommended to avoid these effects [3].

To address overwater image stitching issues, a geometry-based framework involving georeferenced metadata files, which were successfully processed using Erdas Imagine Software [27]. Their published Python code offers a reproducible solution. Alternatives include flying at higher altitudes or ensuring terrestrial features are visible in images to increase keypoint detection [52]. External interferences such as surrounding structures [11] and bottom reflectance in shallow waters [23] were also problematic, often leading to overestimation of chl-a due to aquatic vegetation [53]. No standard correction method currently exists for these optically complex waters. Such errors can mask or exaggerate HAB intensity, resulting in inaccurate risk assessments for both drinking water sources and recreational waterbodies. Turbidity further complicates UAV-based monitoring; however, highly turbid waters tend to reflect more in the red spectrum rather than green, allowing for possible pre-model exclusion [8]. To address these challenges, sensor packages including shortwave infrared (SWIR) could offer improved detection in future applications [28].

### 5.2. Regulatory Considerations for UAV Deployment

The Federal Aviation Administration (FAA) regulates UAV operations in the U.S., classifying them as either “recreational” or “commercial.” Scientific research typically falls under the “commercial” category, requiring compliance with Part 107 regulations, including Part 107 certification via an aeronautical knowledge test [54]. These rules help ensure the safety of researchers and other airspace users. International UAV regulations differ widely and may involve specific operational limits, privacy protections, or restrictions in sensitive areas [10]. For instance, the FAA prohibits UAV flights in U.S. National Parks without pre-approval and restricts certain UAV models in designated zones [55]. Researchers must coordinate with local authorities and remain aware of applicable restrictions. UAV laws are continually evolving. As of 16 September 2023, FAA regulations mandate that all UAVs in the U.S. be equipped with Remote ID, which broadcasts identifying information and flight data [56]. Public safety agencies may request this information when needed.

Beyond the U.S., several other national authorities have established regulatory frameworks for UAV operations. In the U.S. and all the jurisdictions discussed below, drones weighing more than 250 g must be registered with the relevant aviation authority. In the European Union, the European Union Aviation Safety Agency (EASA) governs UAV use, requiring remote pilots to obtain competency certificates (A1/A3 or A2) depending on operational risk [57]. The United Kingdom Civil Aviation Authority (CAA) similarly requires UAV registration and both a Flyer ID and Operator ID, obtained through an online competency test [58]. In Canada, Transport Canada requires either a Basic or Advanced Pilot Certificate depending on proximity to people and airspace class [59]. Australia’s Civil Aviation Safety Authority (CASA) requires either operator accreditation or a Remote Pilot License for commercial use [60]. In South Korea, it is required to hold a remote pilot license and carry mandatory liability insurance of at least 150 million KRW [61]. A comparative summary of registration, certification, and insurance requirements across multiple jurisdictions is provided in Table 3. Overall, understanding and proactively addressing both domestic and international UAV regulations is essential for safe, legal, and successful deployment in HAB-related research [62].

### 5.3. Pre-Flight Planning and Operational Considerations

Effective UAV-based research for HAB monitoring requires thoughtful planning across several domains: UAV and sensor choice, site characteristics, flight parameters, weather, and data processing needs [10,36,55]. The UAV must offer sufficient flight stability, payload capacity, GPS functionality, and compatibility with desired sensors and attachments [32]. Custom modifications should be tested in advance, as they may impact flight performance. Sensor compatibility is also crucial and is discussed in depth in Section 3.2. Site characteristics such as study area size and local air traffic impact flight planning. Larger areas or complex shapes may require waypoint optimization, sufficient image overlap (60–80%), and consideration of battery life and data coverage [41,52]. Pilots must review restricted zones and coordinate with authorities to ensure compliance [55].

Altitude selection influences both battery life and image resolution. While higher flights cover more area, they reduce image detail. Ground Sampling Distance (GSD), typically ranging from 3–20 cm in reviewed studies, should match research goals and equipment capabilities [7,14,41]. Test flights can help identify the optimal balance. Weather is another critical factor. Wind, rain, low visibility, or extreme temperatures can compromise UAV performance and data quality [3,55]. If wind is present but within limits, angling flights to offset drag may preserve battery life [63]. Although many UAVs are water-resistant, flying in rain is discouraged [27]. Lastly, reliable ground control systems (GCS) are essential for safe operation and data management. Ground control points (GCPs) and GNSS improve georeferencing accuracy but may be impractical over water [64]. In such cases, alternative georeferencing methods should be used [10], and GCS compatibility with UAV and sensor systems must be confirmed. For translation into public health practice, officials must be trained in these operational requirements and provided with the appropriate UAV platforms, sensors, and data management tools to ensure monitoring can directly support timely advisories and protective measures.

### 5.4. Future Research and Technology Directions

Across studies, a common recommendation is to standardize UAV imagery processing workflows, as accurate surface reflectance remains difficult to obtain [12,19]. Future research should improve methods to reduce interference and correct for seabed influence in shallow or optically complex waters [23], as well as address atmospheric distortion [17]. Model validation is another area for advancement. Studies emphasized the need for larger datasets and integration of lab-based analyses with UAV imagery [13,26]. Future work should also examine the impact of suspended solids and algal species composition on model accuracy [22] and evaluate model transferability across waterbodies with varying eutrophication levels [9,33]. Incorporating weather data into modeling efforts may improve prediction of surface and subsurface water quality parameters [29]. Continued UAV and sensor development is also critical [7,27,30], and widespread adoption of lightweight hyperspectral sensors may enable spectral library development for improved HAB detection.

Beyond these methodological refinements, frontier approaches such as artificial intelligence, machine learning, and swarm robotics present additional opportunities to expand UAV-based HAB monitoring into predictive, multi-platform systems with direct relevance for public health protection. Deep learning architectures such as convolutional neural networks and long short-term memory models have already outperformed traditional regression approaches in forecasting cyanobacteria abundance and toxin concentrations by leveraging nonlinear relationships among meteorological, hydrodynamic, and nutrient drivers [65]. However, model performance often declines when applied outside the training domain, underscoring the need for multi-season datasets and standardized analytical pipelines [66]. To date, these approaches have been applied mainly to satellite datasets. Extending these to UAV imagery, with its finer spatial and temporal resolution, could improve predictive modeling of bloom dynamics and enable earlier, more targeted public health advisories.

In parallel, multi-platform swarm concepts are beginning to emerge. A UAV–Unmanned Surface Vehicle (USV) framework has demonstrated real-time aerial bloom detection using a Local Binary Pattern segmentation algorithm, with georeferenced coordinates transmitted to a USV performing targeted removal using an electrocoagulation–flotation reactor [67]. Similarly, a dual-UAV deployment paired a sensor-equipped platform with automated water samplers and real-time data transmission with a second UAV performing multispectral imaging and GIS mapping [68]. Although the UAVs did not communicate directly, their complementary roles illustrate how aerial platforms can partition tasks in future swarm-based frameworks. Together, these studies indicate that UAVs are best positioned to act as aerial coordinators in future work, providing wide-area detection while directing complementary platforms for targeted sampling or treatment—an approach that offers more practical public health benefits than UAV-only deployments.

## 6. Conclusions

This review synthesized recent advancements in UAV-based monitoring of HABs, offering practical recommendations and highlighting opportunities for improving public health decision-making. UAVs enhance HAB detection through high-resolution, timely data and complement existing methods such as in situ sampling, satellite imagery, and automated buoy monitoring. By bridging spatial and temporal data gaps, UAVs support multi-scale monitoring approaches that improve situational awareness. Despite challenges—such as weather constraints, overwater image stitching, and spectral interference—ongoing technological improvements and cost reductions are making UAV-based research increasingly viable. Key conclusions from this analysis include:

- Sensor trade-offs: RGB offers low-cost screening, while multispectral and hyperspectral sensors enable more quantitative HAB metrics.
- Data quality hinges on processing: Reflectance retrieval, software selection, and robust validation are essential for reliable outputs.
- Operational and regulatory factors matter: Flight planning, georeferencing, and compliance directly influence applicability.
- Direct public health value: UAV data can strengthen early warning systems, inform water treatment operations, and guide recreational health advisories.

Ultimately, integrating UAV-based monitoring into routine public health practice can strengthen early warning systems, guide drinking water protections, and support timely recreational advisories, ensuring that communities are better protected from the health risks posed by harmful algal blooms.

## Figures and Tables

**Table 1:** Comparative, platform-specific advantages and limitations of UAV designs for HAB monitoring.

	Fixed Wing	Rotorcraft	VTOL
Advantages:	Longer flight times	Perform closer analysis	Vertical take-off and landing
	Survey larger areas	Vertical take-off and landing	Longer flight times
	Carry heavier payloads	Higher spatial resolution	Survey larger areas
		More stable in high winds	
		Can be automated	
Disadvantages:	Difficult takeoff and landing	Shorter flight times.	Expensive
	Low maneuverability		User control limited
	Inability to hover		Altitude requirement for transition to occur

**Table 2:** Spectral indices used for distinguishing HAB from all studies reviewed. Only pre-defined indices were included in this table. If multiple researchers used the same index, all were cited under “reference”.

Index	Formula	Reference
Normalized Difference Vegetation Index (NDVI)	(NIR − Red)/(NIR + Red)	[3,9,14,27,30]
Normalized Green Red Difference Index (NGRDI)	(Green − Red)/(Green + Red)	[13]
Normalized Green Blue Difference Index (NGBDI)	(Green − Blue)/(Green + Blue)	[13]
Green Leaf Index (GLI)	(2 × Green − Red − Blue)/(2 × Green + Red + Blue)	[13]
Excess Green (EXG)	2 × Green − Red − Blue	[13]
Cyanobacteria Index (CI)	CI = −SS (681)	[15]
The Color Producing Algorithm (CPA-A)	Bio-optical inversion model	[15]
Surface Scum Index (SSI)	SSI = ((NIR) − (VIS)/(NIR) + (VIS))	[15]
Kab 1	1.67 − 3.94 × ln(Blue) + 3.78 × ln(Green)	[9,27]
Surface Algal Index (SABI)	(NIR − Red)/(Blue + Green)	[9,27]
KIVU	(Blue − Red)/Green	[9,27]
Normalized Difference Chlorophyll Index (NDCI)	(RE − Red)/(RE + Red)	[9]
2BDA_1 (2 band algorithm)	NIR/Red	[9]
2BDA_2 (2 band algorithm)	RE/Red	[9]
3BDA_1 (3 band algorithm)	(Red^−1^ − RE^−1^) × NIR	[9]
3BDA_MOD (3 band algorithm modified)	Red^−1^ − RE^−1^	[9]
B3B1 (normalized index)	(Green − Blue)/(Green + Blue)	[9]
GB1 (Simple ratio)	Green/Blue	[9]
GR (Simple ratio)	Green/Red	[9]
Normalized Difference of Red Edge (NDRE)	(NIR − RE)/(NIR + RE)	[27,30]
Difference Vegetation Index (DVI)	NIR − Red	[30]
Ratio Vegetation Index (RVI)	NIR/Red	[30]
Blue Normalized Difference Vegetation Index (BNDVI)	(NIR − Blue)/(NIR + Blue)	[27]
Fluorescence Line Height (FLH Blue)	Green − (Red + (Blue − Red))	[27]
SHI Index	(e^Red^ − e^NIR^)/(e^Red^ + e^NIR^)	[27]

**Table 3:** Comparative summary of UAV regulatory requirements across selected regions.

Region/Authority	Pilot Certification	Insurance Required	Potential Issues for HAB Monitoring	Reference
USA (FAA)	Part 107 certificate	Not required by federally	Remote ID adds cost; restricted zones limit sites	[54,55,56]
EU (EASA)	A1/A3 or A2 competency	Mandated in several EU member countries	Insurance varies; cross-border ops complex	[57]
UK (CAA)	Flyer ID + Operator ID	Required for commercial ops	Higher premiums for over-water work	[58]
Canada (Transport Canada)	Basic/Advanced certification	Not required federally	Advanced cert. needed near facilities	[59]
Australia (CASA)	Accreditation or Remote pilot license	Not required federally	Remote pilot license adds cost, exemptions limited	[60]
South Korea (MOLIT)	Remote pilot license	Mandatory (≥150 M KRW)	High insurance cost; strict coastal permits	[61]

## Data Availability

No new data were generated or analyzed in this study. All data presented are derived from previously published sources as part of this review. Data sharing is not applicable.

## Supplementary Materials

The following supporting information can be downloaded at https://www.mdpi.com/article/10.3390/toxins17100475/s1. Table S1. Summary of studies utilizing UAVs for HAB monitoring from 2017–present. Implications for public health of each are discussed [3,7,8,11,12,13,14,15,16,17,18,19,20,21,22,23,24,25,26,27,28,29,30,31,32,33].

## Abbreviations

The following abbreviations are used in this manuscript:

CAA	Civil Aviation Authority
CASA	Civil Aviation Safety Authority
CC	Colored Compounds
CDOM	Colored Dissolved Organic Matter
CNN	Convolutional Neural Network
COST	Cosine of the Solar Zenith Angle
DVI	Difference Vegetation Index
EASA	European Union Aviation Safety Agency
EBS	Ensemble-Based System
FAA	Federal Aviation Administration
fDOM	Fluorescent Dissolved Organic Matter
GCP	Ground Control Point
GCS	Ground Control Station
GNSS	Global Navigation Satellite System
GPS	Global Positioning System
GSD	Ground Sampling Distance
HAB	Harmful Algal Bloom
HSV	Hue Saturation Value
NGBDI	Normalized Green-Blue Difference Index
NGRDI	Normalized Green-Red Difference Index
NDRE	Normalized Difference Red Edge Index
NDVI	Normalized Difference Vegetation Index
NIR	Near Infrared
PC	Phycocyanin
RE	Red Edge (Spectral Band)
RGB	Red Green Blue (Camera)
RMSE	Root Mean Square Error
Rrs	Remote Sensing Reflectance
RRMSE	Relative Root Mean Square Error
R^2^	Coefficient of Determination (Statistical term)
SDD	Secchi Disk Depth
SHI	Spectral Harmonic Index
SWIR	Shortwave Infrared
TN	Total Nitrogen
TP	Total Phosphorus
TSS	Total Suspended Solids
UAV	Unmanned Aerial Vehicle
UAS	Unmanned Aircraft System
USA	United States of America
USV	Unmanned Surface Vehicle
