# Supplementary material for: Harmful Algal Bloom Monitoring with Unmanned Aerial Vehicles: Tools, Challenges, and Public Health Implications
Source: Toxins (Basel). 2025 Sep 24;17(10):475. doi: 10.3390/toxins17100475 (PMC12567913; doi:10.3390/toxins17100475)
Supplement: Supplementary file 1 [file toxins-17-00475-s001.zip › toxins-3838344-SI.pdf]

# Harmful Algal Bloom Monitoring with Unmanned Aerial vehicles: Tools, Challenges, and Public Health Implications

## Supplementary Information

**Table S1.** Summary of studies utilizing UAVs for HAB monitoring from 2017-present. Implications for public health of each are discussed.

| Location | UAV Platform(s) | UAV Attachment(s) | Summary | Potential Public Health Application | Study |
|---|---|---|---|---|---|
| Mexico City, Mexico | DJI Phantom 3 | RGB camera | Aimed to show the potential of using *in situ* and UAV aerial remote-sensing data to analyze the temporal and spatial scale of cyanobacteria in Chapultepec Lakes. | The main activities on the lake are recreational, such as rowing and boat recreation, so a tool is needed that can handle the spatial distribution of HABs. | [11] |
| Kingston, Canada | Custom octorotor | Sony ILCE-6000 | Exhaustively tested how UAV hardware settings affect quality of the data captured. UAV was then deployed to evaluate if UAVs are a viable tool for early and timely cyanobacteria detection. | Timely, flexible, and cost-effective tool for monitoring of HABs using novel algorithm for separating cyanobacteria from other photosynthesizing organisms. | [7] |
| Weitou Bay, China | LT-150 TOPRS Technology | AvaSpec-dual spectroradiometer | UAV assessment of a phytoplankton (Phaeocystis globosa) bloom in an estuary using hyperspectral sensors. | Weitou bay is full of fishing and aquaculture activities. HABs negatively impact human health and the health of local and regional | [12] |

| | | | | | |
|---|---|---|---|---|---|
| | | | | economies, so a tool is needed for accurately quantifying HABs. | |
| Jiangsu province, China | DJI Inspire 1 | DJI X3 RGB camera | Green algae were identified with an UAV. A biomass estimation model was proposed for green algae biomass based on Sentinel-2A image (S2A) and UAV images. | For 10 consecutive years the world's largest green tide disaster has been occurring in the South Yellow Sea, causing nearly 10 billion RMB in economic and ecological losses, so an early warning model is needed. | [13] |
| Changwon County, South Gyeongsang Province, Republic of Korea | FireFLY6 BirdsEyeView Aerobotics | Micasense RedEdge | This study utilized remote sensing techniques using an UAV with multispectral sensor to monitor the Nakdong River. | Sincheon river experiences severe algal blooms every years effecting nearby communities, researcher sought to use UAV on body of water too small to be monitored by satellite | [14] |
| Ohio, USA | Custom quadcopter & hexacopter | Ocean Optics STS Hyperspectral Vis-NIR Spectroradiometer | Deployed two different UAV platform configurations, utilizing Ocean Optics STS spectroradiometers, to measure optical properties of river and lake water for monitoring HAB. | Rapid deployment of this system, low initial cost, high quality of data, ability to take measurements without disturbing water's surface, and low operational costs make UAVs a useful tool for | [15] |

| | | | | | |
|---|---|---|---|---|---|
| | | | | water resource managers. | |
| Namhae, South Korea | Custom blimp | RGB camera | An UAV with a camera was connected to the ground by a rope to detect, determine velocity, and determine movement direction of HABs. | Real time monitoring tool for HABs allowing the location, movement velocity, and movement direction of HABs to be determined around a water desalination plant. | [16] |
| Saemangeum seawall area, South Korea | DJI Inspire 2 | Micasense RedEdge | Developed a new chl-a estimation algorithm based on multispectral camera signals. | A chl-a algorithm, more accurate than others discussed in previous research, was developed in this study, and could be used for chl-a monitoring to improve public health. | [17] |
| Ottowa County, Oklahoma | ATI AgBot | Micasense RedEdge | Sought to create different predictive algorithms of trophic state for optical and non-optical water quality indicators in an oligotrophic system and a eutrophic system using images from an UAV equipped with a multispectral sensor. | UAV imagery used to create statistical models for optical and non-optical water quality properties, indicating UAVs could be a useful tool for water resource managers around the world. | [18] |
| Nagasaki, Japan | DJI Matrice 200 & custom | RGB camera & 3 bottle | An early detection system for HABs using an UAV that locates anomalies | Risk level of HAB is ranked as alert or caution based on reference | [19] |

| | | | | | |
|---|---|---|---|---|---|
| | quadcopter named Akabot II | sampling mechanism | in sea surface reflectance, then an aerial water sampling UAV collects a water sample. | values, this notification is sent to water resource managers and saved in cloud-based system and used for real-time notification. | |
| Central and Eastern Bulgaria | DJI Mavic Pro | RGB Camera | The first use of aerial observations by a drone as an additional means for choosing sampling points during field studies of HABs in Bulgaria. | Ability to speed up detection and reduce sampling efforts while enabling valuable information to be gathered on HABs. | [20] |
| Daejeon, South Korea | DJI Matrice 600 Pro | Nano Hyperspec hyperspecrtal imaging sensor | Generated a vertical pigment-concentration profile with a portable sensor and utilized UAV-based surface reflectance to develop an improved bio-optical remote sensing method. | Daechung reservoir supplies water to surrounding cities for domestic and industrial use, so monitoring is crucial for preserving public health. | [21] |
| Tolo Harbour, Hong Kong | DJI Phantom 4 | RGB camera | For the first time, this study demonstrates the use of an UAV to quantitatively map surface water chl-a distribution in coastal waters from a low altitude utilizing an estimation model for chl-a concentration. | Blooms in this area can lead to massive fish kills within the coastal fish farms and can lead to beach closures. Research provides a tool to quantitively map HABs. | [22] |
| Iberian Peninsula, Spain | Octocopter Atyges FV8 | Micasense RedEdge | Researchers sought to combine the affordability, stability, quality | Since 2000, the reservoir has maintained a drinking water | [9] |

| | | | | | |
|---|---|---|---|---|---|
| | | | and continuity of ESA and NASA missions (satellite) with the flexibility and spatial resolution provided using UAV platforms. | supply service for households and facilities in the municipality. The reservoir also has a river park and recreational areas. Tool developed to combine satellite and UAV monitoring. | |
| Southern England, United Kingdom | DJI Phantom 3 Pro | Sentera Multispectral Sensor | Aimed to evaluate the ability of UAVs equipped with multispectral sensors for inferring the spatial distribution of chl-A concentration and turbidity in surface waters. | Provides the ability to estimate the spatial distribution of chl-a concentrations. | [23] |
| Nakdong River, South Korea | DJI Phantom 4 | RGB camera | Aimed to develop a practical and rapid countermeasure to HABs by identifying HAB prone regions with acoustic doppler current profiler measurements and visually inspecting HAB prone regions with UAV. | The Chilseo WTP is in operation downstream providing water to Daegu and Busan, and is affected by these blooms, harmful microcystins could affect the public water drinking system. Began developing rapid countermeasure for HABs. | [24] |
| Maldonado, Uruguay | DJI Phantom 4 Pro | Parrot Sequoia multispectral (Parrot SA) | Used a UAV equipped with multispectral sensor to estimate chl-a and cyanobacteria concentrations using many | De Los Cisnes Lagoon supplies drinking water to more than 95 percent of the fixed population. This study shows it is possible to identify | [3] |

| | | | | | |
|---|---|---|---|---|---|
| | | | different band combinations. | cyanobacteria among other photosynthetic organisms using an UAV equipped with a four-band multispectral camera. | |
| Lake Balaton, Hungary | Mavic 2 Enterprise | Thermal dual camera & BD2Vision LaQuinta multipsectral | Developed a new methodology to determine algal concentrations in lakes, utilizing a UAV equipped with a multispectral camera. | The quick-process measurements obtained in this study can be done as frequently as required with a markedly lower budget. | [25] |
| Suolongtan Lake, China | DJI Inspire 1 | Zenmus X3 optical camera | The seasonal shoreline and eutrophication of a desert lake were monitored using an UAV and water sampling during three crop growth stages. | Desert lakes cover a small portion of the desert landscape but provide important ecological services and benefits to residents. UAVs can be a tool for better understanding the eutrophication of these water resources. | [26] |
| Iowa, USA | DJI Inspire 1 | Micasense Rededge-3 | Proposed the use of a multiscale, multimethod approach using near-range imaging, bioassay, and chromatography methods to best capture bloom dynamics. | Sought to generate multi-platform framework for predicting microcystins in water sources. | [27] |

| | | | | | |
|---|---|---|---|---|---|
| Quebec, Canada | Custom hexacopter | Hyperspectral Pika 2 & Pika NIR | Presented a development effort for a regional/local EBS-based model for chl-a estimates in freshwater bodies that can be run on both data acquired by an UAV and Sentinel-2 data. | An algorithm is developed that can be applied to both sentinel-2 data and UAV imagery; Satellite data could be used for analysis at regional scale while water managers could utilize UAVs at a local scale. | [8] |
| Maryland, USA | DJI Phantom 4 Pro | Micasense RedEdge-MX | Presents a comparison of four approaches to remove sun glint and surface reflected light that can be applied to UAS remote sensing to derive water quality parameters such as chl-A concentration. | Improved water quality monitoring of coastal and inland water bodies to effectively track trends, identify and mitigate pollution sources, and discern potential human health risks. | [28] |
| Geum, South Korea | Matrice 600 Pro | Nano Hyperspec hyperspectral imaging sensor | Applied a deep neural network model to monitor the vertical distribution of chl-A, PC, and turbidity using drone borne hyperspectral imagery, in-situ measurements, and meteoroidal data. | Deep learning models could be used to better understand and predict vertical migration of HABs. This could allow for more informed early warning systems. | [29] |
| Haizhou Bay, China | Ecodrone UAS-8 m | Micasense RedEdge-M & FireFly 8 s camera | Aimed to establish an algorithm model of biomass based on multispectral imaging data, which could be used for estimating *P. yezoensis* biomass | Accurate estimations of macroalgae production are economically important for providing food, medicine, | [30] |

| | | | | | |
|---|---|---|---|---|---|
| | | | in an accurate, high-throughput, and non-destructive way. | cosmetics, and biofuel. | |
| Ohio, USA | DJI Inspire 2 | Impinging device & optical particle counter | An Airborne DROne Particle-monitoring System (AirDROPS) was developed and used to monitor, collect, and characterize airborne particles over two HABs. | Lake spray aerosols can contain toxins, which can be transported inland to communities, negatively impacting public health. UAVs could be a new tool for evaluating the toxicity of HAB aerosols. | [31] |
| Ohio & Virginia, USA | DJI Phantom 4 | RGB camera & water sampling tube | Utilized drone-based water sampling methods to characterize cyanotoxins, PC, and nutrients in three freshwater lakes in the United States with active HABs. | Demonstrates the potential for drone-based water sampling technologies to be used by public health and water quality experts to provide critical and timely information for regulatory decisions and health advisories. | [32] |
| Daechung Lake, South Korea | Matrice 600 Pro | Nano Hyperspec hyperspectral imaging sensor | Evaluated the potential of deep learning models to estimate biomass pigments (i.e. chl-a and PC) and accessory pigments (i.e. lutein, fucoxanthin, and zeaxanthin). | Deep learning model could accurately analyze algal phenomenon quantitatively and qualitatively when using UAV imagery spatial information input of HABs. | [33] |
